# What was new at the 2023 American Diabetes Association meeting?

**DOI:** 10.1111/1753-0407.13447

**Published:** 2023-08-11

**Authors:** Zachary Bloomgarden

**Affiliations:** ^1^ Department of Medicine, Division of Endocrinology, Diabetes and Bone Disease Icahn School of Medicine at Mount Sinai New York New York USA

An approach is to review the late‐breaking abstracts. In principle, these abstracts recognize novel, important research developments of sufficient scientific importance to merit special consideration after the standard abstract deadline. Some were indeed of interest and, taken together, the following summary of just a portion of these abstracts provides a view of the myriad areas of development in diabetes. For much additional information, see the Close Concerns Knowledgebase giving day‐by‐day summaries of the entire meeting.*

## 1 MICRORNA

MicroRNA (miRNA) are small, single‐stranded, noncoding RNA molecules involved in RNA silencing and post‐transcriptional regulation of gene expression by binding to a complementary sequence in a coding RNA to prevent transcription. It is estimated that miRNAs regulate approximately 30% of the protein‐coding genes in humans, and their roles are likely to be extremely important in understanding the metabolic abnormalities of diabetes. Tryggestad et al (159‐LB) found that increased miR‐155 and miR‐130b and decreased miR‐126 were associated with decrease in the C‐peptide oral disposition index in participants in the Treatment Options for Type 2 Diabetes in Adolescents and Youth (TODAY) study, as potential markers of β cell dysfunction in youth‐onset type 2 diabetes (T2D). Redling et al (160‐LB) analyzed miR from 365 TODAY participants at 0, 2, 5, 8 and 10 years; 68% of participants developed ≥1 microvascular complications, which were associated with increased levels of miR‐122 and miR‐221, and decreased miR‐let7g and miR‐130a, consistent with roles of endothelial dysfunction. Chen et al (22‐LB) profiled perivascular adipose tissue‐derived exosome miR in the db/db T2D rodent model, suggesting potential mediators of adipose tissue inflammation and browning.

## 2 RENAL DISEASE

Balogh et al (10‐LB)^†^ administered fluvoxamine to streptozotocin‐diabetic rats, showing improvement in glomerular filtration rate (GFR) and reduction in proteinuria, renal fibrosis, and expression of in hypoxia‐induced inflammatory mediators, suggesting that sigma‐1 receptor agonists (RAs), which include fluvoxamine, donepezil, and neurosteroids such as progesterone and testosterone, may have renoprotective benefits. Yin et al (11‐LB) randomized 239 persons with T2D, glycated hemogloblin (HbA1c) microalbuminuria, estimated GFR (eGFR) ≥60 mL/min/1.73 m^2^, and HbA1c ≤ 9%, to Huangkui capsule, an extract from the flowers of Abelmoschus manihot, or to losartan 100 mg daily for 24 weeks, reporting that eGFR decreased by 4 mL/min/1.73 m^2^ and the urine albumin‐creatinine ratio decreased by 79 and 57 mg/g, respectively, suggesting that this plant extract, widely used for renal disease in China,^1^ might be considered for treatment of diabetic kidney disease (recognizing that the complex governmental requirements for medication approval make this not a trivial undertaking, see).^2^

## 3 EYE DISEASE

Sidhu et al (207‐LB) analyzed development of primary open‐angle glaucoma among 18 440 persons with diabetes, finding that metformin use was associated with a 67% lower likelihood of development of glaucoma after multivariate correction. An et al (27‐LB) studied 306 patients with diabetic retinopathy, finding that adiponectin, leptin, and fatty acid‐binding protein 4 are higher in those with sight‐threatening retinopathy than in those with mild retinopathy, and that among 110 persons with mild nonproliferative retinopathy those with elevated levels of the three adiponectins had greater likelihood of developing sight‐threatening retinopathy. Gonzalez et al (25‐LB) treated 103 persons with nonproliferative diabetic retinopathy for 24 weeks with placebo versus APX3330, a small molecule inhibitor of redox factor 1, which activates transcription factors including nuclear factor kappa B and hypoxia‐inducible factor 1 alpha; although the primary end point of diabetic retinopathy stage improvement was not met, there were trends to less frequent worsening of vision.

## 4 DIABETIC NEUROPATHY

Wang et al (203‐LB) enrolled 14 908 T2D patients from 24 provinces in China in 2017, finding 68% prevalence of peripheral neuropathy, characterized as mild, moderate, and severe in 48%, 33%, and 19%, respectively; neuropathy increased with age and with lower income and education level. Armstrong et al (29‐LB) randomized 96 patients with diabetic foot wounds to weekly treatment with the Medaxis water‐jet wound debridement device versus standard sharp debridement along with wound dressing and offloading, finding 65% versus 42% healing at 16 weeks with fewer wound‐related adverse events, suggesting a relatively “low‐tech” approach that might be useful for this major diabetes‐related issue.

## 5 LIFESTYLE

Bahman and colleagues (41‐LB) found that higher accelerometer‐measured physical activity among 69 normal‐weight persons was associated with lower matrix metallopeptidase 9 and higher levels of toll‐like receptor 2‐positive circulating monocytes. Odegaard et al (44‐LB) reported continuous glucose monitoring data for 166 persons with T2D randomized to diet beverages versus unflavored sparkling or flat bottled water, 24 oz daily, finding no effect on time in range or in glycemic variability after 24 weeks in both arms. Heianza et al (48‐LB) randomized T2D persons treated with metformin or not requiring medication to a high‐protein/low‐carbohydrate diet with 30% low starch carbohydrate, 30% protein, and 40% fat or to a control diet containing 55% carbohydrate, 15% protein, and 30% fat, finding similar weight loss in both groups but a significantly greater reduction in HbA1c from baseline of 7.7%–6.6% with the lower carbohydrate diet, compared with the decrease from 7.7% to 7.2% with the control diet. Mohan et al (46‐LB) randomized 38 Asian Indian adults with T2D to adding sucralose to coffee, tea, and fruit juices or to a control group, finding no change in gut microbiome but a significant reduction in weight and body fat.

## 6 CARDIOVASCULAR DISEASE (CVD)

Ke and Fend (24‐LB) studied 2912 persons with T2D, 360 with left ventricular hypertrophy (LVH); controlling for hypertension and coronary disease, retinopathy was associated with 1.3‐fold increase and nephropathy with a 2.1‐fold increase in likelihood of LVH, suggesting microvascular disease as a potential mediator. Hong et al (281‐LB) reported a strong relationship for all‐cause and for CVD mortality with pericardial fat volume on cardiac computed tomography in 6785 participants without pre‐existing cardiovascular disease from the Multi‐Ethnic Study of Atherosclerosis (MESA), although the associations were attenuated by adjustment for demographics, CV risk factors, income, and waist‐to‐hip ratio.

Zareini et al (200‐LB) compared CV benefit of dual glucagon‐like peptide‐1 receptor agonist (GLP‐1RA) and sodium‐glucose cotransporter‐2 inhibitors (SGLT2i) in 87 201 patients from the Danish nationwide registries followed from start of dual second‐line T2D treatment in 2010–2021; Of these patients, 14 831 received GLP‐1RA plus SGLT2i; 20 417 received GLP‐1RA and a dipeptidyl peptidase inhibitor, sulfonylurea, or thiazolidinedione (DPP4/SU/TZD); 22 803 received SGLT2i and DPP4/SU/TZD; and 29 150 received dual DPP4/SU/TZD. GLP‐1RA plus SGLT2i was the only combination associated with reduction in heart failure, and both GLP‐1RA/SGLT2i and GLP‐1RA with one of DPP4/SU/TZD were associated with significant reduction in major adverse cardiac events and in all‐cause mortality. The same group (201‐LB) showed that dual SGLT2i and DPP4/SU/TZD were associated with 32% reduction in the risk of chronic kidney disease and that GLP‐1RA plus SGLT2i was associated with 48% reduction in risk of eGFR decrease >50%. Nguyen et al (103‐LB) used a clinical database with 8.5‐month follow‐up of >40 000 persons with T2D who had CVD and had developed COVID; in a propensity score matching analysis, the 20% who were treated with a SGLT2i or GLP‐1RA had 12% lower likelihood of hospitalization and 10% lower likelihood of intensive care unit admission.

## 7 INCRETIN‐BASED THERAPIES

Several alternatives to weekly injection of these agents are being developed. Je et al (93‐LB) described in in vitro and nonhuman primate studies of ID110521156, a small‐molecule oral selective GLP‐1 RA potentially suitable for treatment of T2D and obesity. Wu et al (95‐LB) administered another potential oral small molecule GLP‐1RA, HRS‐7535, to healthy volunteers for 28 days, reporting mild nausea and vomiting, with weight loss suggesting suitability for clinical use. Seol and Kim (78‐LB) described a 28‐day sustained‐release semaglutide preparation without an initial burst and without a lag phase in rat and minipig animal studies.

To et al (72‐LB) described results of T2D treatment with DD01, a dual GLP‐1/glucagon receptor agonist, in 6 T2D persons, resulting at 4 weeks in a 52% reduction in magnetic resonance imaging‐measured hepatic fat content versus 2.8% reduction with placebo. Zhu et al (79‐LB) presented results of a 206‐person study of a novel once‐weekly GLP‐RA, ecnoglutide at 1.2, 1.8, or 2.4 mg doses, compared with liraglutide 3 mg daily for 26 weeks, leading to weight loss of 11.5%, 11.2%, 14.7%, and 8.8%, respectively, with similar gastrointestinal symptoms in all groups; one participant receiving 1.2 mg ecnoglutide and one in the liraglutide group developed acute cholecystitis. Olsen et al (92‐LB) administered the amylin analog ZP8396 versus placebo to 56 nondiabetic persons, showing dose‐related weight loss and a 10‐day half‐life, suggesting suitability for weekly administration with the potential for combined administration with incretins.

Amamoo et al (86‐LB) presented 12‐month data from a claims and electronic medical record database of 398 T2D persons treated with oral semaglutide, with HbA1c decreasing from 8.8 to 7.8%, and with body mass index decreasing from 35.2 to 34.1 kg/m^2^. The same group (87‐LB) described 12‐month results among 1926 persons with T2D receiving injectable semaglutide, finding similar decrease in HbA1c from 8.9 to 7.6% and body mass index decrease from 36.5 to 35 kg/m^2^.

## 8 SODIUM‐GLUCOSE COTRANSPORTER‐2 INHIBITORS (SGLT2I)

In a study of the potential role of SGLT2i in reducing renal hypoxia as a mechanism of renal protection, Hesp et al (7‐LB) administered ertugliflozin versus placebo to 20 persons with T2D in a randomized, double‐blind crossover study, with metformin and an angiotensin receptor blocker also given, finding that eGFR and tubular Na transport decreased, the latter correlating with a decrease in renal O2 consumption. Wen (244‐LB) administered dapagliflozin or saline to nondiabetic mice, finding blood pressure lowering without change in heart rate; immunohistochemical studies showed SGLT2 expression in specific regions involved in autonomic control; dapagliflozin administration increased expression in these regions of neuronal c‐Fos, an indirect marker of neuronal activity, suggesting a potential effect of SGLT2i in sympathetic inhibition contributing to its CV benefits.

Kuchay et al (100‐LB) treated 250 noncritically ill, hospitalized T2D patients with dapagliflozin plus basal‐bolus insulin versus insulin alone, finding ketone levels of 0.7 versus 0.30 mmol/L, with 6 patients receiving the SGLT2i developing severe ketonemia, but not DKA; glycemic control was similar in the two groups, suggesting that (if otherwise indicated) initiating such treatment is feasible during hospitalization. Laffel et al (164‐LB) treated 157 T2D patients ages 10–17 with empagliflozin, linagliptin, or placebo, finding that 26‐week likelihoods of HbA1c <7.0% or change from baseline ≤ − 0.5% in the three groups were 51.9%, 30.8%, and 28.3%, suggesting SGLT2i to be appropriate for T2D youth.

## 9 OTHER THERAPIES

Rodriguez et al (LB‐91) presented results of a small, controlled trial of BMF‐219, an oral inhibitor of menin, which is involved in transcription of multiple genes. Menin inhibits gene transcription; in the study BMF‐219 was administered for 4 weeks, either with food in one 10‐person cohort or without food in a second 10‐person cohort, with the latter group appearing to show effective HbA1c reduction from baseline of 8.1 by 1.0% at 12 weeks, and with increased C‐peptide at 4, 8, and 12 weeks and increased homeostasis model assessment of beta‐cell function, suggesting durable improvement. There is a complex interrelationship between prolactin and menin, and Wang (231‐LB) reported an in vitro study of pancreatic ductal cells exposed to prolactin, showing evidence of differentiation into islet‐like cells; in an in vivo study of diabetic mice transplanted under the kidney capsule with the prolactin‐stimulated pancreatic ductal cells, glucose lowering with serum C‐peptide elevation was observed.

Zhang et al (96‐LB) administered the glucokinase activator dorzagliatin to T2D persons, showing improvements in beta‐cell function with the oral minimal model study. Roman et al (293‐LB) showed that in isolated islets from T2D donors dorzagliatin lowered the threshold for glucose‐stimulated insulin secretion, suggesting that the glucose dependency of this agent may reduce the likelihood of hypoglycemia in clinical use. Han et al (297‐LB) showed restoration of glucose‐stimulated insulin secretion with dorzagliatin in islets of high‐fat diet‐induced diabetic mice.

## 10 GLUCOSE SENSING AND INFUSION

Lu et al (118‐LB) reported use of the SynerGTM combined insulin infusion cannula and glucose sensor in 15 type 1 diabetes participants using a Medtronic 780G insulin pump, finding that 99% of data pairs with capillary glucose and with arterialized blood were in zones A and B of the classical error grid, albeit with mean absolute relative difference (MARD) of 13.5%. Geiser et al (119‐LB) presented preliminary accuracy analysis of a noninvasive subdermal laser‐scanned glucose sensing prototype in 18 adults with type 1 diabetes, showing that 99% of data pairs with continuous glucose monitoring devices were in zones A and B of the Clarke error grid, with MARD of 19%. Hirsch and Navon (130‐LB) reported preliminary results of the GWave device (Hagar, Israel) using radiofrequency to detect capillary glucose in nondiabetic persons, showing 96% and 97% in zone A of the Clarke error grid compared to venous and capillary blood, respectively. Pfützner et al (133‐LB) described an osmotic‐pressure based continuous glucose sensor with MARD of 9.8% in comparison to capillary glucose measurement; the authors suggest the device might allow >6–12 month duration of use with wireless energy and data transfer.

O'Connor et al (125‐LB) reported analysis of Dexcom continuous glucose monitoring data from 268 hospitalized persons; 17% were determined at least once to have a false glucose reading <70 mg/dL, but, excluding such, 11% had at least one episode of glucose<54 mg/dL. Whether this technology will prove useful in in‐patient management remains to be seen.

Cohen et al (107‐LB) noted that insulin pump canula failure are due to inflammation from trauma during insertion, as well as to insulin aggregates and other impurities in the infusate; failure rates based on unexplained hyperglycemia were 37% at 3 days with the Medtronic Mio 30 infusion set, whereas a new extended version had a 6‐day failure rate of 30%.

## Endnotes

* Day 1 – https://www.closeconcerns.com/knowledgebase/r/9bfb57f9; day 2‐ https://www.closeconcerns.com/knowledgebase/r/410eb12a; day 3 – https://www.closeconcerns.com/knowledgebase/r/c8b04230; day 4 – http://www.closeconcerns.com/knowledgebase/r/d14e83f9.

^†^
Rather than citing each reference, the reference designation is given. All are available at https://diabetesjournals.org/diabetes/issue/72/Supplement_1.
